# Insular cortex Hounsfield units predict postoperative neurocardiogenic injury in patients with aneurysmal subarachnoid hemorrhage

**DOI:** 10.1002/acn3.51926

**Published:** 2023-10-18

**Authors:** Yitong Jia, Fa Lin, Runting Li, Yu Chen, Jun Yang, Heze Han, Ke Wang, Kexin Yuan, Yang Zhao, Junlin Lu, Tu Li, Zhaobo Nie, Yunfan Zhou, Guangzhi Shi, Youxiang Li, Yuanli Zhao, Xiaolin Chen, Shuo Wang

**Affiliations:** ^1^ Department of Neurosurgery Beijing Tiantan Hospital, Capital Medical University Beijing China; ^2^ China National Clinical Research Center for Neurological Diseases Beijing China; ^3^ Center of Stroke, Beijing Institute for Brain Disorders Beijing China; ^4^ Beijing Key Laboratory of Translational Medicine for Cerebrovascular Disease Beijing China; ^5^ Department of Neurosurgery Peking University International Hospital Beijing China; ^6^ Department of Neurosurgery West China Hospital, Sichuan University Sichuan China; ^7^ Beijing Shunyi Hospital Shunyi Teaching Hospital of Capital Medical University Beijing People's Republic of China; ^8^ Department of Critical Care Medicine Beijing Tiantan Hospital, Capital Medical University Beijing China

## Abstract

**Objective:**

Our study aims to investigate the association between the Hounsfield unit (Hu) value of the insular cortex (IC) during emergency admission and the subsequent occurrence of post‐operative neurocardiogenic injury (NCI) among patients afflicted with aneurysmal subarachnoid hemorrhage (aSAH).

**Methods:**

Patients baseline characteristics were juxtaposed between those with and without NCI. The significant variables were incorporated into a multivariable stepwise logistic regression model. Receiver operating characteristic (ROC) curves were drafted for each significant variable, yielding cutoff values and the area under the curve (AUC). Subgroup and sensitivity analyses were performed to assess the predictive performance across various cohorts and ascertain result stability. Propensity score matching (PSM) was ultimately employed to redress any baseline characteristic disparities.

**Results:**

Patients displaying a right IC Hu value surpassing 28.65 exhibited an escalated risk of postoperative NCI upon confounder adjustment (*p* < 0.001). The ROC curve eloquently manifested the predictive capacity of right IC Hu in relation to NCI (AUC = 0.650, 95%CI, 0.591–0.709, *p* < 0.001). Further subgroup analysis revealed significant interactions between right IC Hu and factors such as age, history of heart disease, and Graeb 5–12 score. Sensitivity analysis further upheld the results' significant (*p* = 0.002). The discrepancy in NCI incidence between the two groups, both prior (*p* < 0.002) and post (*p* = 0.039) PSM, exhibited statistical significance. After PSM implementation, the likelihood of NCI displayed an ascending trend with increasing right IC Hu values, from the Hu_1_ cohort onward, receding post the Hu_4_ cohort.

**Conclusion:**

This study definitively establishes an elevated right IC Hu value in the early stages of emergency admission as an autonomous predictor for ensuing NCI subsequent to aSAH.

## Introduction

Aneurysmal subarachnoid hemorrhage (aSAH) represents a critical neurosurgical cerebrovascular emergency, where the spontaneous rupture of an intracranial aneurysm culminates in hemorrhage within the subarachnoid space.^1^, ^2^, ^3^, ^4^ A discernible towards diminished incidence is intricately linked to public health interventions and lifestyle adaptations.^5^ Capitalizing on the progressive advancements in cerebrovascular surgical diagnostics and therapeutics, the previously formidable case fatality rate of aSAH, hovering around 40%, has witnessed substantial reduction.^6^ However, a considerable cohort of patients may present an extensive array of postoperative complications due to underlying pathophysiologic mechanisms.^7^, ^8^

Contemporary investigations pertaining to the sequela of aSAH have predominantly concentrated on the realm of delayed cerebral ischemia (DCI).^9^, ^10^ As scientific exploration delves deeper into the intricate interplay between the brain and the heart, cardiac complications have gained prominence within the aSAH domain. Notably, these complications, particularly neurocardiogenic injury (NCI), have demonstrated associations with DCI and adverse outcomes.^11^, ^12^, ^13^ Furthermore, pertinent preoperative relevant cardiovascular biomarkers such as cardiac troponin I have exhibited connection with unfavorable postoperative outcomes.^11^, ^12^, ^14^, ^15^, ^16^

Antecedent research has illuminated the indispensable role of the insular cortex (IC) within the central automatic network in regulating of the cardiovascular system.^17^, ^18^ To be specific, IC stimulation triggers activation of the sympathetic nervous system, engendering manifestations such as tachycardia and heightened catecholamine release, factors that may precipitate myocardial necrosis.^19^, ^20^, ^21^, ^22^, ^23^, ^24^ Thus, impairment of the IC could potentially contribute to the genesis of NCI subsequent to aSAH. Furthermore, the severity of aSAH appears to correlate positively with the magnitude of NCI.^21^

Rapid processing of perfusion and diffusion (RAPID), an image post‐processing software, has the capacity to automatically assess the Alberta Stroke Program Early CT score (ASPECTS) through computation of mean Hounsfield unit (Hu) values within the middle cerebral artery (MCA) territory.^1^, ^25^ Hounsfield units (Hu) represent a quantitative density metric employed by radiologists for interpreting CT images.^26^ Previous researches have elucidated the prognostic significance of Hu values concerning DCI and clinical outcomes among aSAH patients.^27^, ^28^ Nevertheless, studies on the potential correlation between IC Hu values and cardiac events in aSAH patients remain limited.

In the pursuit of discerning whether the Hu values can function as robust indicators of cardiac injury, facilitating judicious preemptive monitoring and intervention, our study endeavors to scrutinize the conceivable interrelation between IC Hu values upon admission and the manifestation of NCI in patients with aSAH.

## Methods

### Study design and population

**Figure 1:**
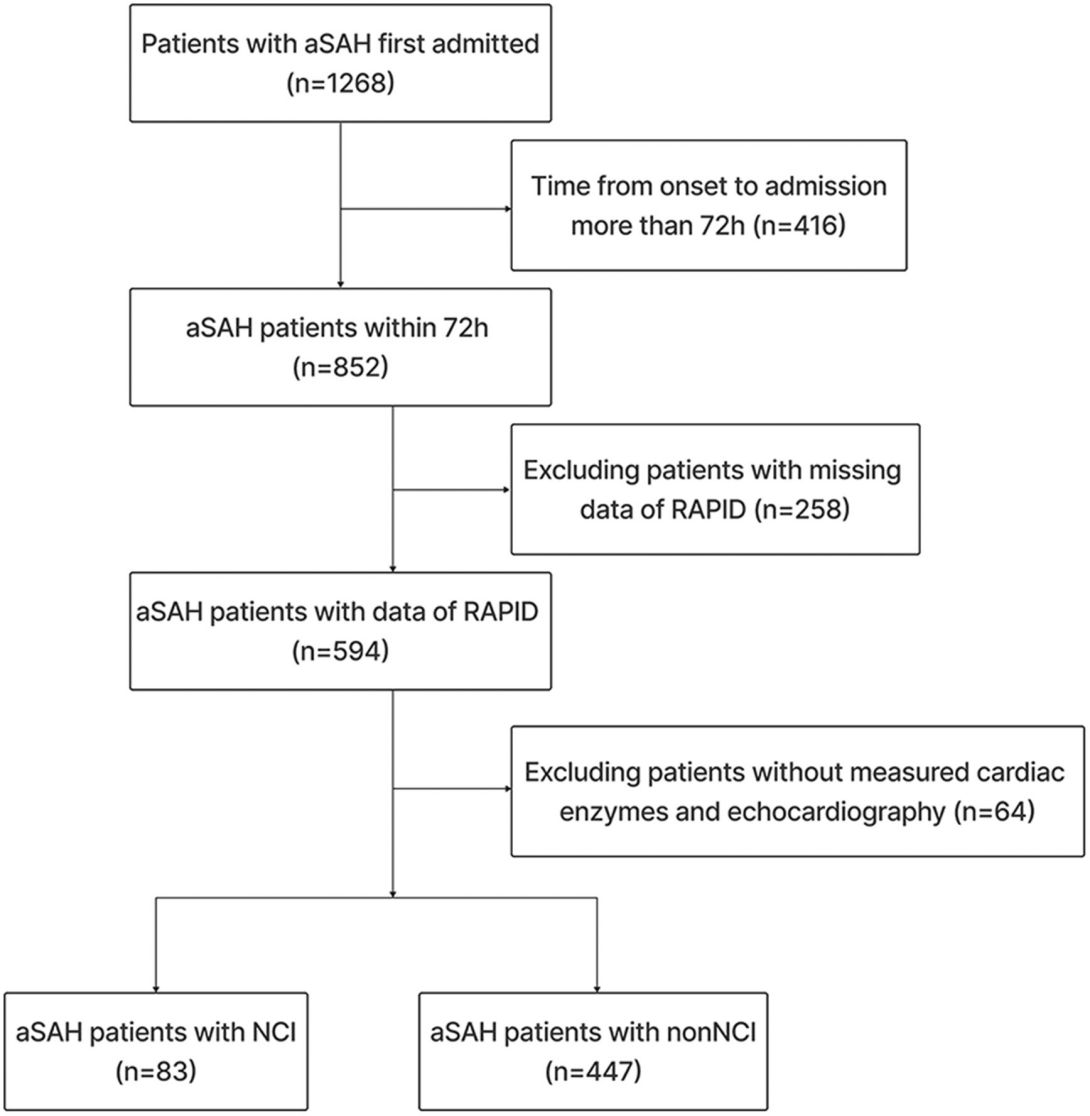
Flow chart showing inclusion/exclusion of patients.

Patients diagnosed with aSAH and who underwent assessment of cardiac enzymes and echocardiography within a 72‐h window from the onset of bleeding were deemed eligible for participation in this single‐center, retrospective, observational study, carried out at Beijing Tiantan Hospital spanning the period from January 2019 to September 2022, as illustrated in Fig. 1. Within this study framework, patients afflicted with aSAH were diagnosed employing cranial computed tomography (CT), lumbar puncture, CT angiography (CTA), or digital subtraction angiography (DSA). The stipulated inclusion criteria encompassed: (1) attainment of an age surpassing 18 years; (2) admission of aSAH patients within 72‐h interval following symptom onset; (3) cases necessitating emergency admission; (4) presence of solitary aneurysm; (5) application of either surgical clipping or endovascular intervention as treatment modalities. Conversely, the set of exclusion criteria encompassed: (1) instances of subarachnoid hemorrhage arising from nonaneurysmal causes or vascular malformations; (2) documented history of prior SAH or intracerebral hemorrhage (ICH); (3) a medical background involving cerebral surgical interventions; (4) manifestation of bilateral mydriasis or other enduring cerebral injuries at the point of admission; (5) instances of incomplete data, particularly in terms of radiological CT scans.

Approval from the Institutional Review Board at Beijing Tiantan hospital, specifically under reference KY 2021‐008‐01, was duly secured prior to the initiation of this study. Informed consent conscientiously obtained from all individual participants or their legally authorized representatives. The research was conducted in strict accordance with the principles in the Declaration of Helsinki. It is noteworthy that the management of all patients adhered diligently to established guidelines.^5^, ^29^

### Clinical data collection

We extracted patient data during the acute phase from our electrical medical records, encompassing: (1) demographic particulars: age, gender, and medical history (hypertension, hyperlipidemia, diabetes mellitus, current smoking, and drinking status, and heart disease); (2) clinical condition: aneurysm location and the maximal diameter; World Federation of Neurosurgical Societies (WFNS) grade; modified Fisher scale (mFS); Graeb score; Subarachnoid Hemorrhage Early Brain Edema (SEBES) score; existence of acute hydrocephalus; (3) treatment modality (surgical clipping or endovascular treatment).

### Imaging acquisition and radiological data

**Figure 2:**
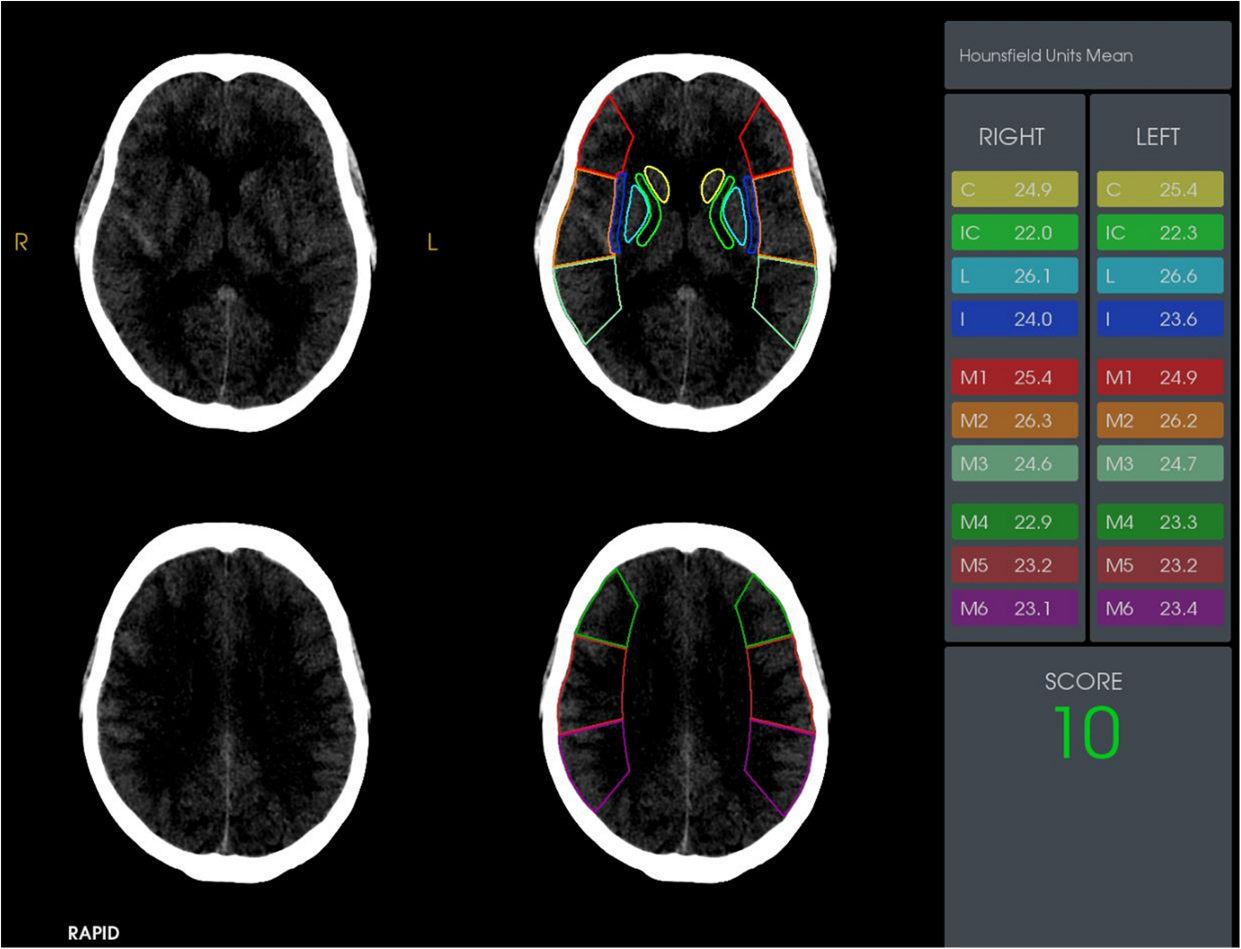
Imaging acquisition of RAPID‐ASPECTS.

The image dataset underwent processing via the RAPID post‐processing software, meticulously designed for ischemic penumbra identification.^30^ Furthermore, the ASPECTS scoring system was employed for an intricate early infarct analysis.^31^ ASPECTS predominantly encompasses two CT tiers: the basal ganglia and the body of the lateral ventricle. Distinct colorations are employed to demarcate distribution zones for each branch of the MCA, vividly visible in Fig. 2. Utilizing RAPID ASPECTS, mean CT values for MCA distribution regions can be automatically computed, enabling ASPECT score assessment within the specific IC region of interest (ROI) designated for this study.

### Outcome assessment

The primary objective of our research is to ascertain the frequency of postoperative NCI. NCI was delineated by one or more of the subsequent criteria: (1) elevated serum myocardial enzyme levels: cardiac troponin I > 1.0 ng/mL; creatine kinase myocardial isoenzyme >1%; (2) aberrant echocardiography presentation: abnormal ventricular wall motion. The aforementioned presentation may be accompanied by brain natriuretic peptide >100 pg/mL, and conceivable electrocardiographic alterations such as T‐wave inversion and QT‐interval prolongation. Profound myocardial injury can precipitate a deterioration in left ventricular systolic and diastolic function.^13^, ^21^, ^22^, ^23^, ^24^, ^32^, ^33^

### Statistical analysis

Prior to analysis, the distribution pattern of each continuous variable underwent assessment via visual examination using Q‐Q plots or histograms, and numerically through the Shapiro–Wilk test. Additionally, the equivalence of variance between two random groups was evaluated using Levene's test. Variables that exhibited a near‐normal distribution and comparable variances were subjected to independent *t*‐tests or ANOVA tests, with results presented as mean ± standard deviation (SD). Conversely, variables failing to meet these assumptions were subject to the Mann–Whitney *U*‐test, with outcomes presented as medians along with interquartile ranges (IQR). Categorical variables were scrutinized using the chi‐squared test or Fisher's exact test, depending on anticipated frequencies.

Subsequently, risk factors associated with NCI, having attained a *p*‐value < 0.05 during univariable analysis, were subjected to multivariable logistic regression. This step aimed to rectify confounding biases and ascertain the independent impact of each variable on the outcome. Results were expressed as odds ratios (ORs) accompanied by 95% confidence intervals (95% CIs). These findings were then visualized via a graphical representation illustrating adjusted predicted probabilities concerning NCI occurrence in individual patients, correlated against the principal independent predictor, namely the right IC Hu.

A receiver operating curve (ROC) analysis was performed to determine the threshold mean CT Hu value, subsequently establishing the area under the curve (AUC) as an indicator of each factor's predictive potential. Additionally, subgroup analyses, depicted through forest plots, were executed to explore the predictive efficacy of right IC Hu in subpopulations exhibiting distinct characteristics from the overall cohort. Given the Hu value representation within the MCA regions as reflected in ASPECTS, a supplementary analysis was pursued, excluding cases of MCA aneurysms. This was carried out to ascertain whether elevated right IC Hu remained an independent predictor in aSAH patients with aneurysms situated in alternate regions.

To delve deeper into relationship between the admission right IC Hu and in‐hospital NCI complications, the study partitioned the population based on a predetermined cutoff value for right IC Hu. Measures were taken to address potential confounding biases arising from baseline characteristic imbalances through propensity score matching (PSM), utilizing a match tolerance of 0.02 and a 1:1 ratio. The incidence of in‐hospital NCI complications was treated as a binary variable in the matched pairs and evaluated via the chi‐squared test.

Lastly, the range of right Hu values was divided into quartiles and median intervals. The changing trend in the likelihood of NCI occurrence with increasing Hu intervals was depicted through a bar line chart.

All statistical analyses aforementioned were performed utilizing SPSS Statistics 26.0 (IBM, Armonk, New York, USA), R software (Version 4.1.2), and GraphPad Prism 9.3.1 (GraphPad Software Inc., San Diego, CA, USA). Statistical significance was defined at two‐tailed *p*‐value < 0.05.

## Results

### Patients' baseline characteristics

A cohort totaling 530 patients emerged as the focal point of our investigation, have been meticulously selected from a pool of 1268 aSAH cases. The dichotomy between two distinct groups, predicated on the presence of NCI, is expounded upon in Table 1. Noteworthy disparities materialized among patients with and without NCI. Specifically, those afflicted with NCI displayed advanced age (59.4 ± 11.2 vs. 54.8 ± 11.4, *p* = 0.001) and exhibited a heightened propensity for antecedent cardiovascular maladies (15.7% vs. 5.4%, *p* = 0.001). Concurrently, the NCI cohort manifested a severe clinical presentation of aSAH at admission, underscored by escalated WFNS scores (34.9% vs. 18.6%, *p* = 0.001), augmented mFS ratios (77.1% vs. 56.6%, *p* < 0.001), elevated Graeb scores (18.1% vs. 4.3%, *p* < 0.001), and an increased incidence of acute hydrocephalus (57.8% vs. 35.8%, *p* < 0.001). Additionally, the NCI group exhibited a higher frequency of posterior circulation aneurysms (18.1% vs. 9.6%, *p* = 0.023). Moreover, discernible elevations in the values of right IC Hu (30.1 ± 3.1 vs. 28.7 ± 2.5, *p* < 0.001) and left IC Hu (29.3 ± 2.6 vs. 28.5 ± 2.9, *p* = 0.019) were observed in the NCI cohort relative to their non‐NCI counterparts. Notably, a larger proportion of NCI‐affected patients underwent endovascular interventions (60.2%) compared to their non‐NCI counterparts (60.2% vs. 38.7%, *p* < 0.001).

**Table 1:** Baseline characteristics of patients with aSAH according to NCI.

Patient characteristics	NCI	nonNCI	*p‐*value
No. of patients (*n* = 530)	83	447	
Demographics			
Age, years; mean ± SD	59.4 ± 11.2	54.8 ± 11.4	0.001
Gender, female, *n* (%)	54 (65.1)	270 (60.4)	0.424
Current drinking, *n* (%)	8 (9.6)	49 (11.0)	0.506
Current smoking, *n* (%)	5 (6.0)	34 (7.6)	0.612
Hypertension, *n* (%)	50 (60.2)	220 (49.2)	0.065
Hyperlipidemia, *n* (%)	4 (4.8)	8 (1.8)	0.088
Diabetes mellitus, *n* (%)	8 (9.6)	22 (4.9)	0.088
History of heart disease, *n* (%)	13 (15.7)	24 (5.4)	0.001
Preoperative condition			
WFNS grade 4–5, *n* (%)	29 (34.9)	83 (18.6)	0.001
mFS grade 3–4, *n* (%)	64 (77.1)	253 (56.6)	<0.001
Graeb score 5–12, *n* (%)	15 (18.1)	19 (4.3)	<0.001
SEBES score 3–4, *n* (%)	40 (48.2)	187 (41.8)	0.282
Acute hydrocephalus, *n* (%)	48 (57.8)	160 (35.8)	<0.001
Imaging index			
Right IC Hu; mean ± SD	30.1 ± 3.1	28.7 ± 2.5	<0.001
Left IC Hu; mean ± SD	29.3 ± 2.6	28.5 ± 2.9	0.019
Posterior circulation aneurysm, *n* (%)	15 (18.1)	43 (9.6)	0.023
Aneurysm size^a^, median (IQR)	5 (4, 7)	6 (4, 8)	0.841
Treatment modality			<0.001
Endovascular treatment, *n* (%)	50 (60.2)	173 (38.7)	
Surgical clipping, *n* (%)	33 (39.8)	274 (61.3)	

aSAH, aneurysmal subarachnoid hemorrhage; Hu, Hounsfield unit; IC, insular cortex; IQR, interquartile range; mFS, modified Fisher; NCI, neurocardiogenic injury; SD, standard deviation; SEBES, subarachnoid hemorrhage early brain edema score; WFNS, World Federation of Neurosurgical Surgeons.

^a^ Unit of measurement: mm.

### Relationship between right IC Hu and admission time, relationship between NCI and admission time

**Figure 3:**
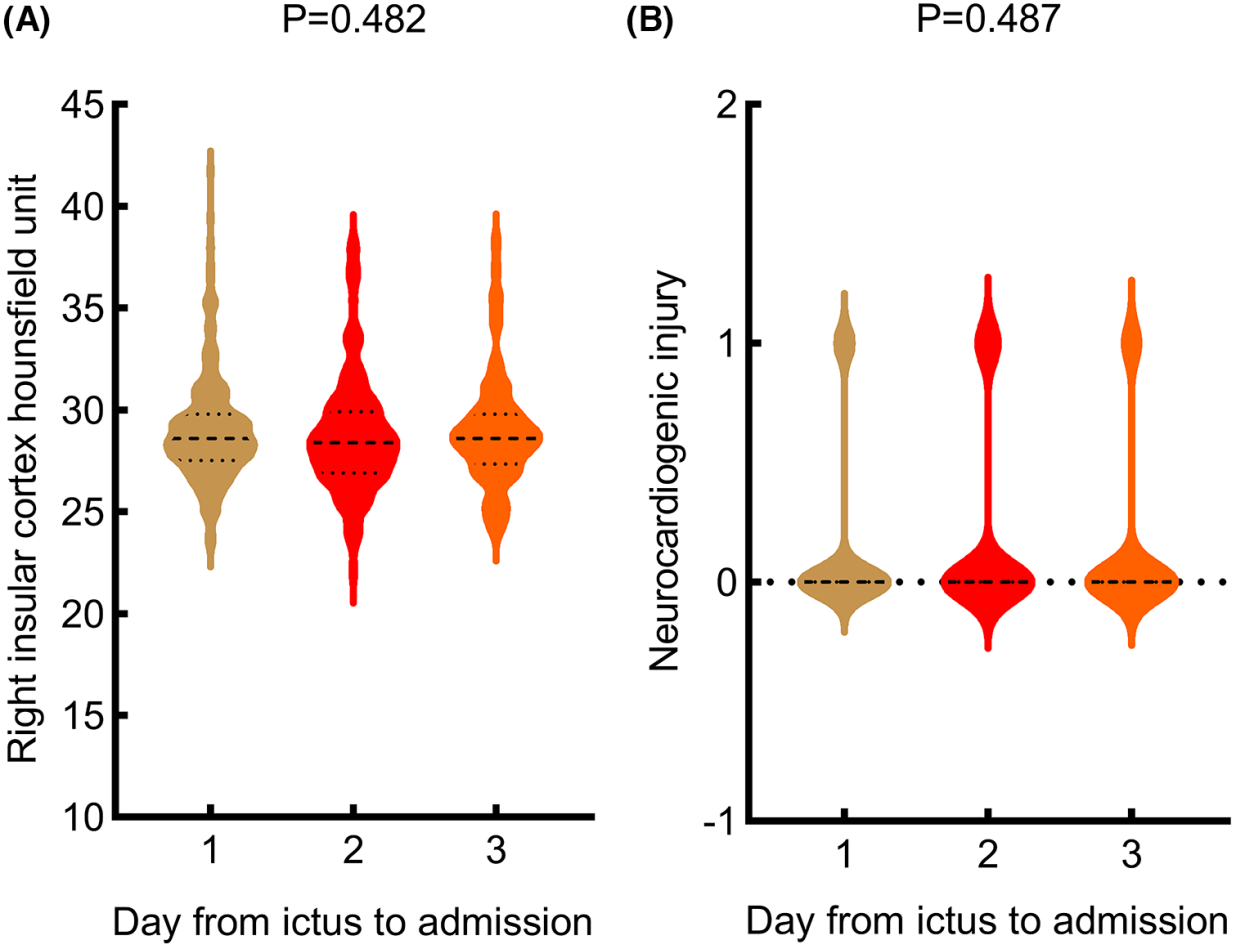
(A) Relationship between right IC Hu and admission time. (B) Relationship between NCI and admission time.

The right IC Hu for patients admitted within 24, 48, and 72 h from aSAH onset were 28.9, 28.7, and 28.9, respectively. None of the patients admitted within these time frames experienced postoperative NCI. There were no significant differences observed in either right IC Hu (*p* = 0.482) or NCI (*p* = 0.487) between the groups (Fig. 3).

### Association of right IC Hu at admission with NCI

**Figure 4:**
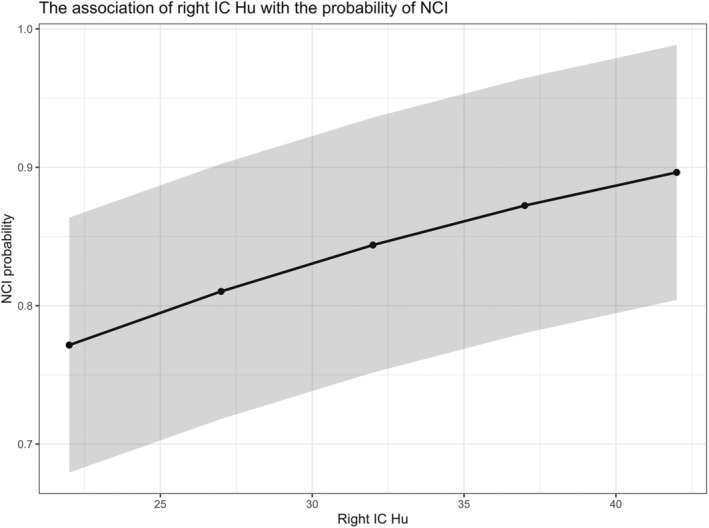
The association of right IC Hu with the probability of NCI. There is an increase in the likelihood of NCI with increase of right IC Hu (adjusted for age, history of heart disease, Graeb 5–12, acute hydrocephalus, treatment modality).

Table 2 illustrates the outcomes of executing stepwise multivariable logistic regression analyses, founded upon significant variables recognized in the univariable regression. Ultimately, a total of six variables manifested in the multivariable output: age, history of cardiac ailments, Graeb score ranging from 5 to 12, acute hydrocephalus, right IC Hu, and endovascular intervention. These factors exhibited independent predictability concerning the incidence of NCI in postoperative scenarios. Figure 4 elaborates upon the adjusted correlation between the right IC Hu and the probability of NCI.

**Table 2:** Multivariable analysis of the relationship between the variables and NCI for cases after operation.

Variables	Univariable			Multivariable		
	OR	95% CI	*p*‐value	OR	95% CI	*p*‐value
Age	1.04	1.02–1.06	0.001	1.03	1.00–1.06	0.023
Gender	0.82	0.50–1.34	0.425			0.167
Hypertension	1.56	0.97–2.52	0.066			0.242
History of heart disease	3.27	1.59–6.73	0.001	2.57	1.14–5.81	0.024
WFNS 4–5	2.36	1.41–3.92	0.001			0.107
mFS 3–4	2.58	1.50–4.46	0.001			0.169
Graeb 5–12	4.97	2.41–10.25	<0.001	3.14	1.38–7.16	0.006
Acute hydrocephalus	2.46	1.53–3.96	<0.001	2.43	1.41–4.18	0.001
Posterior circulation aneurysm	2.07	1.09–3.94	0.026			0.829
Left IC Hu	1.09	1.01–0.17	0.024			0.978
Right IC Hu	1.20	1.11–1.30	<0.001	1.23	1.12–1.34	<0.001
Endovascular treatment	2.40	1.49–3.87	<0.001	2.41	1.42–4.09	0.001

CI, confidence interval; Hu, Hounsfield unit; IC, insular cortex; mFS, modified Fisher; NCI, neurocardiogenic injury; OR, odds ratio; WFNS, World Federation of Neurosurgical Surgeons.

**Figure 5:**
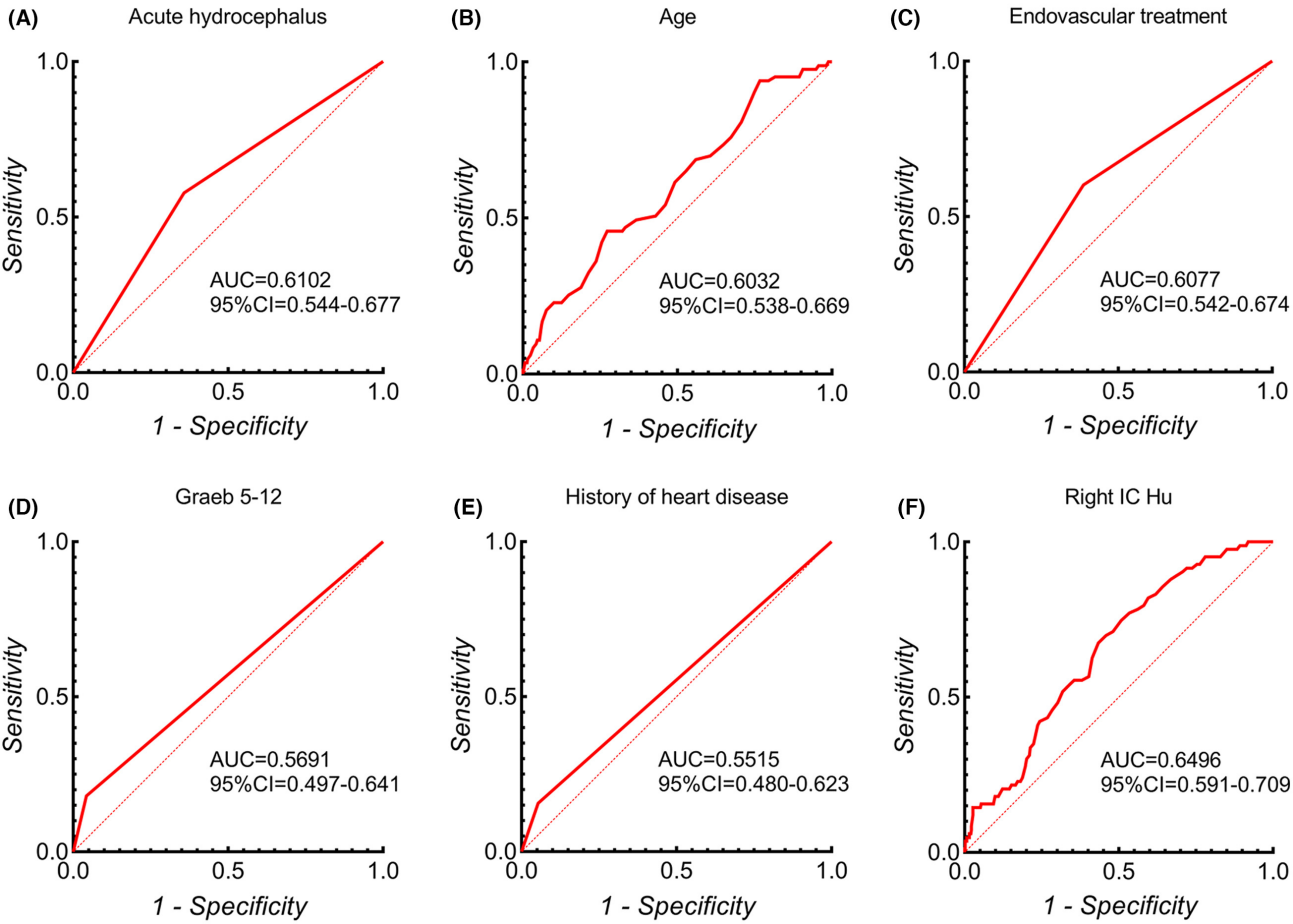
Comparison of AUC values of right IC Hu (F) and other preoperative independent risk factors (A‐E).

The research employed the ROC curve to ascertain the optimal threshold value for the right IC Hu, which materialized as 28.65, as determined by the selection of the maximal Youden index. Pursuant to this threshold, it was ascertained that patients exceeding this right IC Hu value were predisposed to NCI development. Moreover, Fig. 5 portrays the area under the ROC curve accompanied by a 95%CI, along with *p*‐values corresponding to all independent predictors. The outcomes underscored that the right IC Hu stood as the most robust predictor (right IC Hu>28.65, AUC = 0.650, 95%CI, 0.591–0.709, *p* < 0.001).

### Subgroup and sensitivity analysis of the association between right IC Hu and NCI

**Figure 6:**
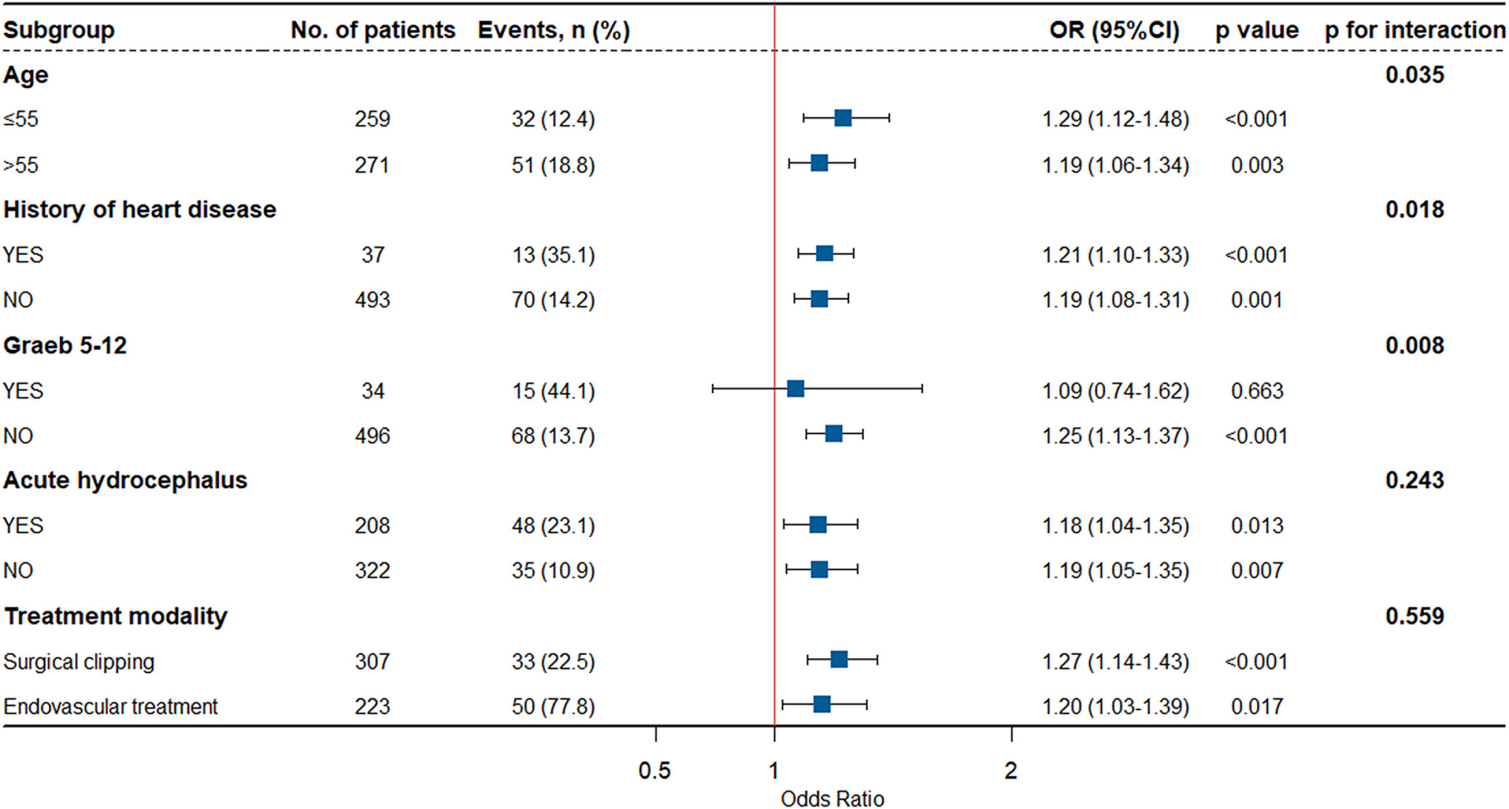
Subgroup analysis of the association between right IC Hu and neurocardiogenic injury.

In Fig. 6, the subgroup analysis unveils a compelling interplay wherein the right IC Hu exhibit noteworthy correlations with age, a history of heart disease, and Graeb scores ranging from 5 to 12. The influence of the right IC Hu on the prevalence of NCI manifests more prominently within the cohort of patients with Graeb 5–12, in stark contrast to those with Graeb 0–4 (*p* for interaction = 0.008). Analogous findings reverberate within the subgroups of patients with a history of heart disease (*p* for interaction = 0.018) and varying age strata (*p* for interaction = 0.035). Conversely, other subgroupings, including those encompassing acute hydrocephalus and treatment modalities, fail to manifest any discernible nexus.

Upon subjecting the analysis to a sensitivity evaluation that excludes cases involving aneurysms in the MCA zone, the outcomes remain notably unaltered. The prognostic potency of the right IC Hu concerning postoperative NCI endures its stability. Even when instances involving MCA aneurysms are excluded, the cohort comprising aSAH patients persistently showcases heightened susceptibility to postoperative NCI with the escalating right IC Hu values (*p* = 0.002) (Table 3).

**Table 3:** Sensitivity analysis after removal of MCA aneurysm cases.

Variables	OR (95%CI)	*p*‐value
Age	1.04 (1.01–1.07)	0.013
Graeb 5–12	2.42 (0.96–6.08)	0.060
Acute hydrocephalus	3.21 (1.68–6.13)	<0.001
Endovascular treatment	2.69 (1.44–5.01)	0.002
History of heart disease	2.60 (1.04–6.50)	0.042
Right IC Hu	1.22 (1.08–1.39)	0.002

CI, confidence interval; Hu, Hounsfield unit; IC, insular cortex; MCA, middle cerebral artery; OR, odds ratio.

### Association of right IC Hu with NCI after PSM

The disparity in NCI incidence between the two groups exhibited statistical significance both prior to (*p* < 0.001) and subsequent to (*p* = 0.039) PSM. Notably, a significant variance was discerned among groups with mFS grade 3–4 following PSM (*p* < 0.001) (Table S1). The special baseline characteristics of the patients with postoperative NCI was listed, as well as the subcategories of each NCI, as detailed in Table S2.

**Figure 7:**
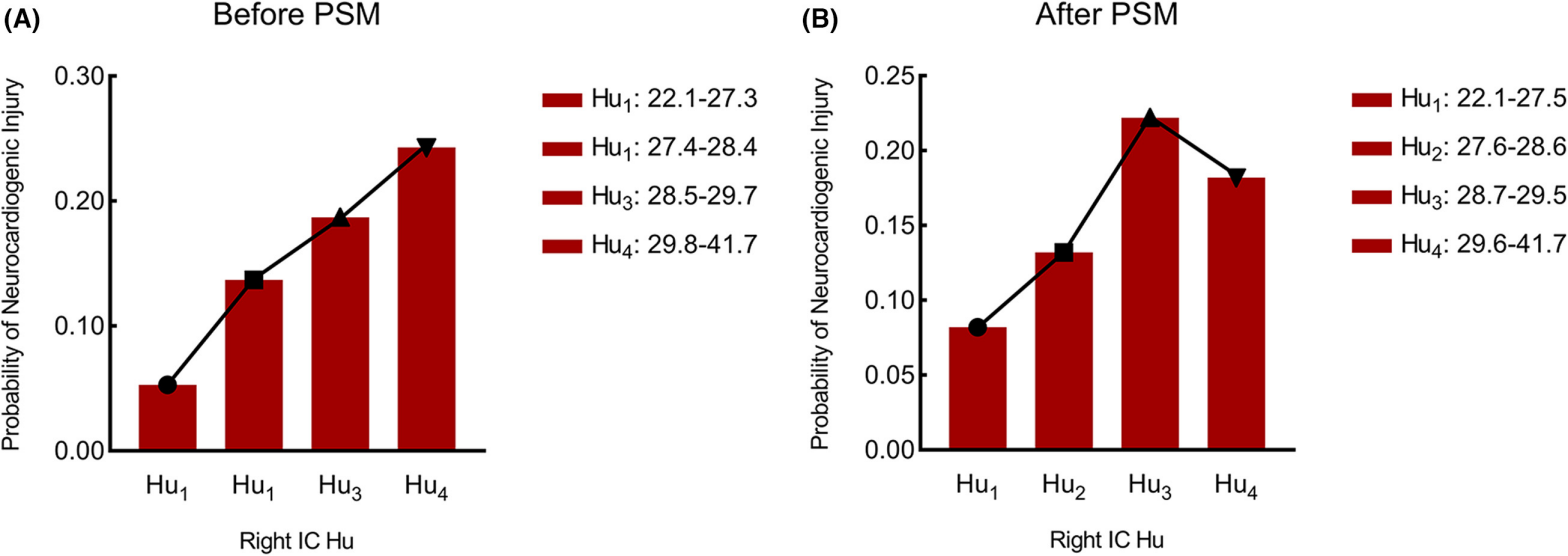
The relationship between right IC Hu and the probability of NCI before PSM (A) and after PSM (B): A line connecting the midpoints of the top edge of each of the four bars chart was added to generate a bar line chart, with the respective interquartile range of Hu as the horizontal axis and the probability of NCI for each interval of Hu as the vertical axis.

To illustrate the outcomes of PSM, a bar chart was crafted, partitioning the respective values of right IC Hu into four intervals (Hu_1_‐Hu_4_) based on quartiles and medians, both before and after the matching procedure (Fig. 7). Pre‐PSM, the likelihood of NCI escalation was concomitant with augmented right IC Hu values, originating from the Hu_1_ range (22.1–27.3) (7/132 [5.3%]) and culminating in the Hu_4_ category (29.8–41.7) (34/140 [24.3%]). Subsequent to PSM, the probability of NCI elevation correlated with heightened right IC Hu values, initiating from the Hu_1_ range (22.1–27.5) (5/61 [8.2%]) and abating beyond the Hu_4_ range (29.6–41.7) (12/66 [18.2%]). No statistically significant differences in probability were observed between contiguous groups.

## Discussion

Our study explores the correlation between IC Hu values and cardiovascular events in aSAH. Employing both multivariable logistic regression and propensity score matching techniques, we meticulously accounted for confounding variables within our sample. Our analyses unveiled a notable association between elevated right IC Hu values, particularly those surpassing a specified threshold, and the frequent occurrence of postoperative myocardial injury among emergent aSAH patients (Fig. S1). Meanwhile, this correlation is independent of the type of intervention and usual risk factors. In a subgroup analysis, we ascertained that the interplay between right IC Hu values and intraventricular hemorrhage wielded a substantial influence on NCI.

While the ASPECTS remains a dependable and uncomplicated scoring system for appraising early ischemic changes within the distribution zones of the MCA, it is crucial to acknowledge the potential variability in interpretations due to divergent levels of expertise among non‐contrast CT observers.^34^ In contrast, the RAPID ASPECTS system harnesses validated machine‐learning algorithms to autonomously identify cerebral regions and compute scores, thereby enabling swift and more harmonious assessment for thrombectomy eligibility (*κ* = 0.9), surpassing the concordance of human readers (*κ* = 0.57 and *κ* = 0.56).^31^

Prior investigative trajectories focusing on the predictive potential of CT Hu values for DCI and clinical outcomes predominantly centered on average Hu values within specific hemorrhage tiers.^27^, ^28^, ^35^ In contradistinction, the Hu value derived from the RAPID ASPECTS software furnishes objective imaging metrics for each distinct zone, facilitating a nuanced evaluation of complications or prognosis. This approach circumvents the inherent subjective bias that could manifest when experts choose the CT plane, rendering it an advanced stride. Remarkably, this marks the inaugural clinical application of mean insular CT Hu value extracted from the RAPID ASPECTS in the realm of aSAH patients management.

The intricate configuration of neural networks presumably exhibits variations contingent upon definitions, specifically reliant on the distinct autonomic outcome measures. However, it consistently perceives the IC as a pivotal constituent, particularly concerning cardiac regulation.^36^, ^37^, ^38^ Extensive research has delved into the mechanisms through which IC impairment may precipitate cardiac injury. Among these, Michiaki Nagai's exploration of Takotsubo syndrome, a transient cardiomyopathy triggered by acute emotional or physical stress, stands prominent.^18^ Earlier studies have substantiated the notion that the cardiovascular system finds governance within the central automatic network (CAN), comprising the IC, anterior cingulate gyrus, and amygdala.^17^ Normally, the CAN maintains a dynamic equilibrium between the sympathetic nervous system (SNS) and the parasympathetic nervous system under physiological conditions. Disruption to either facet of the CAN disturbs the balance of automatic function.^39^

A preceding study has demonstrated that inactivation of the right hemisphere led to an elevation in heart rate and blood pressure's high‐frequency components, while left hemisphere inactivation yielded the converse, especially notable in drug‐refractory epilepsy patients.^19^ By bilaterally stimulating the IC, Oppenheimer SM discovered that right IC stimulation induced tachycardia and pressor effects, whereas left IC stimulation elicited bradycardia and depressor effects.^20^ Ayano Osawa proposed that damage to the right insular cortex, possibly inducing heightened sympathetic nervous system activity, played a role in CAN dysregulation, myocardial injury, escalated brain natriuretic peptide levels, and the incidence of Takotsubo cardiomyopathy.^40^ Consequently, human right IC stimulation escalated sympathetic cardiovascular tone, while left IC stimulation more frequently elevated parasympathetic activity. The exacerbated SNS activity stemming from right IC stimulation might underlie myocardial necrosis. This form of cardiotoxicity following aSAH manifests electrocardiographic alterations, abnormal ventricular wall motion, cardiac insufficiency, and elevated serum myocardial enzyme levels.^21^ Our study highlights that solely the Hu value of the right IC exerts an independent impact on NCI, whereas the left IC Hu value does not exhibit such influence according to univariable analysis. These findings intimate that autonomic control of cardiac activity is lateralized, likely predominantly mediated by the right‐sided IC. Nonetheless, through both univariable and multivariable regression analyses, it was observed that the left IC Hu value bore correlation with other variables. Thus, significant influences of the left IC on the cardiovascular system should not be dismissed.

Upon the occurrence of aSAH, the stimulation of the right IC by blood may indeed be the instigator of sympathetic overactivation, mirroring a comparable phenomenon. Subsequent to aSAH, left ventricular systolic dysfunction correlates with regular myocardial perfusion juxtaposed with abnormal augmentation in sympathetic activity.^23^ In situations of unaltered myocardial perfusion, catecholamines released from sympathetic terminals provoke myocardial necrosis, its extent intricately linked to the severity of aSAH.^21^ Furthermore, a discernible association surfaces between right‐hemispheric IC strokes and sinus tachycardia, escalated plasma levels of norepinephrine, augmented blood pressure, diminished heart rate variability, electrocardiographic abnormalities, and amplified mortality.^38^ It has been posited that instances of right MCA infarction ensuing vasospasm post aSAH impede IC functionality, consequentially inducing heightened sympathetic cardiovascular tone and the cardiac ramifications of the stroke.^37^, ^38^, ^41^

Our research employed myocardial enzymes as pivotal indicator for prognostic discernment. A plethora of antecedent studies expounded upon the correlation between troponin with myocardial impairment following a stroke.^14^, ^16^, ^42^, ^43^, ^44^ Additionally, some researchers have alluded to the concurrence of IC impairment due to stroke with elevated troponin concentrations.^45^, ^46^ Scheitz, J.F. and colleagues delineated that augmented cardiac troponin T levels were independently correlated with heightened stroke severity (*p* = 0.04) and IC involvement (*p* < 0.001).^45^ These findings collectively buttress the assertion that troponin release and IC injury subsequent to aSAH stand as pivotal cues for NCI.

An intriguing phenomenon emerged: the interplay between Graeb 5–12 and the right IC Hu value impacting NCI. Within subgroups, patients with acute hydrocephalus exhibited a notable positive predictive effect on outcomes associated with higher right IC Hu values. In instances of intracerebral hemorrhage, QTc prolongation on ECG seemed associated with IC involvement, the presence of intraventricular blood, and hydrocephalus.^47^ Explorations of structural connectivity in brain networks unveiled significant discrepancies in regional network measures, including IC in hydrocephalus groups, compared to controls.^48^ Building on these pertinent findings, we scrutinized cases involving intraventricular hemorrhage or acute hydrocephalus, leading to relevant speculations. Our hypothesis posited that the proximity between the lateral ventricle and IC could exacerbate stimulation and extrusion to IC in aSAH patients facing intraventricular hemorrhage or acute hydrocephalus.

A representative case was chosen, featuring 78‐year‐old female with aSAH and diagnosed NCI (Fig. S2). This patient, devoid of heart disease history, encountered NCI on the fifth post aneurysm clipping day. A CTA unveiled an anterior communicating artery (AcoA) aneurysm. Her right insular Hu value reached 29.8, contrasting with the contralateral value of 27.9, as indicated by preoperative RAPID‐ASPECTS within 24 h post‐aneurysmal rupture onset. Upon experiencing symptoms like chest tightness, breathlessness, and dyspnea on the fifth day post‐surgery, laboratory tests unveiled elevated CK‐MB levels. Echocardiography further disclosed abnormal left ventricular wall motion and reduced apical motion. Swiftly, a brain–heart combined treatment approach was instituted, and collaborative consultation determined NCI. Subsequent steps encompassed intensive care, oxygen therapy, sedation, fluid intake management, ventricular rate control, and myocardial ischemia reduction. Post‐symptom stabilization, cerebral CT, CTA, and CTP scans detected no rebleeding or hypoperfusion. Ultimately, the patient's condition ameliorated, and cardiac indicators normalized. Hence, meticulous focus on the ICU's ECG monitoring and timely postoperative echocardiography, along with cardiac biomarker assessment around the fifth postoperation day, especially in the IC region indicated by the preoperative emergency CT‐ASPECTS, becomes imperative. These measures, anticipate cardiac function shifts, thereby enhancing postoperative functional prognosis.

Nevertheless, our study bears limitations. First, it is a retrospective, single‐center, observational case–control study, susceptible to recall bias and constrained by a modest sample size. Nonetheless, it is worth noting that this study represents, to the best of our knowledge, the largest cohort of aSAH cases focused on IC Hu values and NCI. Therefore, future prospects necessitate large‐scale, prospective, multicenter studies to authenticate our findings. Second, despite controlling key clinical baselines, potential confounders might persist, necessitating refined designs in future studies to mitigate biases. Third, our analysis centered solely on Hu values from one region, prompting future studies to encompass supplementary imaging indicators for a more comprehensive analysis. Finally, while these differences were statistically significant, the small differences in IC Hu values between the two groups is valid. We posit that this subtle variance might be attributed to the ensuing factors: (1) Unlike the relatively confined cerebral parenchymal hemorrhage, aSAH manifests in a more dispersed manner, with the ROI encompassing the insula presenting a diminutive expanse at the level of the basal ganglia on CT scans. Consequently, the amplitude of Hu value fluctuations within the insula ROI due to multifaceted pathological determinants could be restricted; (2) The precision of the right IC Hu value in prognosticating NCI was utilized to ascertain the threshold for prediction (right IC Hu > 28.65). Given the variable spectrum of the right IC Hu values in our dataset, a larger and heterogeneous populace would be requisite to authenticate these findings in subsequent investigations.

## Conclusion

The study unveiled the early elevation of right insular cortex CT Hu value following emergency admission as an independent predictor of neurocardiogenic injury post aSAH. This CT density‐based prognostication for clinical cardiac complications could substantially inform perioperative management.

## Author Contributions

YTJ, RTL, FL, XLC, and SW brought forward an idea. YTJ, RTL, FL, YC, KXY, JY, HZH contributed to data curation and project administration. ZBN, JLL, YZ, YXL, GZS, XLC contributed to methodology and investigation. YTJ, FL, RTL, TL, YFZ contributed to software. YTJ contributed to writing original draft, validation, visualization. SW contributed to writing review and editing, resources, supervision, and funding acquisition.

## Funding Information

This work was supported by the National Key Research and Development Program of China (Grant No. 2021YFC2501101 and 2020YFC2004701), Bai Qian Wan Talent Plan (Grant No. 2017A07).

## Conflict of Interest Statement

The authors declare that they have no conflicts of personal, financial, or institutional interest in any of the materials or methods used in this study or the findings specified in this paper.

## Supporting information


Figure S1.

Figure S2.

Table S1.

Table S2.

Table S3.

## Data Availability

The data that support the findings of this study are available from the corresponding author upon reasonable request.
