# Supplementary figures and images for: Insular cortex Hounsfield units predict postoperative neurocardiogenic injury in patients with aneurysmal subarachnoid hemorrhage
Source: Ann Clin Transl Neurol. 2023 Oct 18;10(12):2373–85. doi: 10.1002/acn3.51926 (PMC10723248; doi:10.1002/acn3.51926)

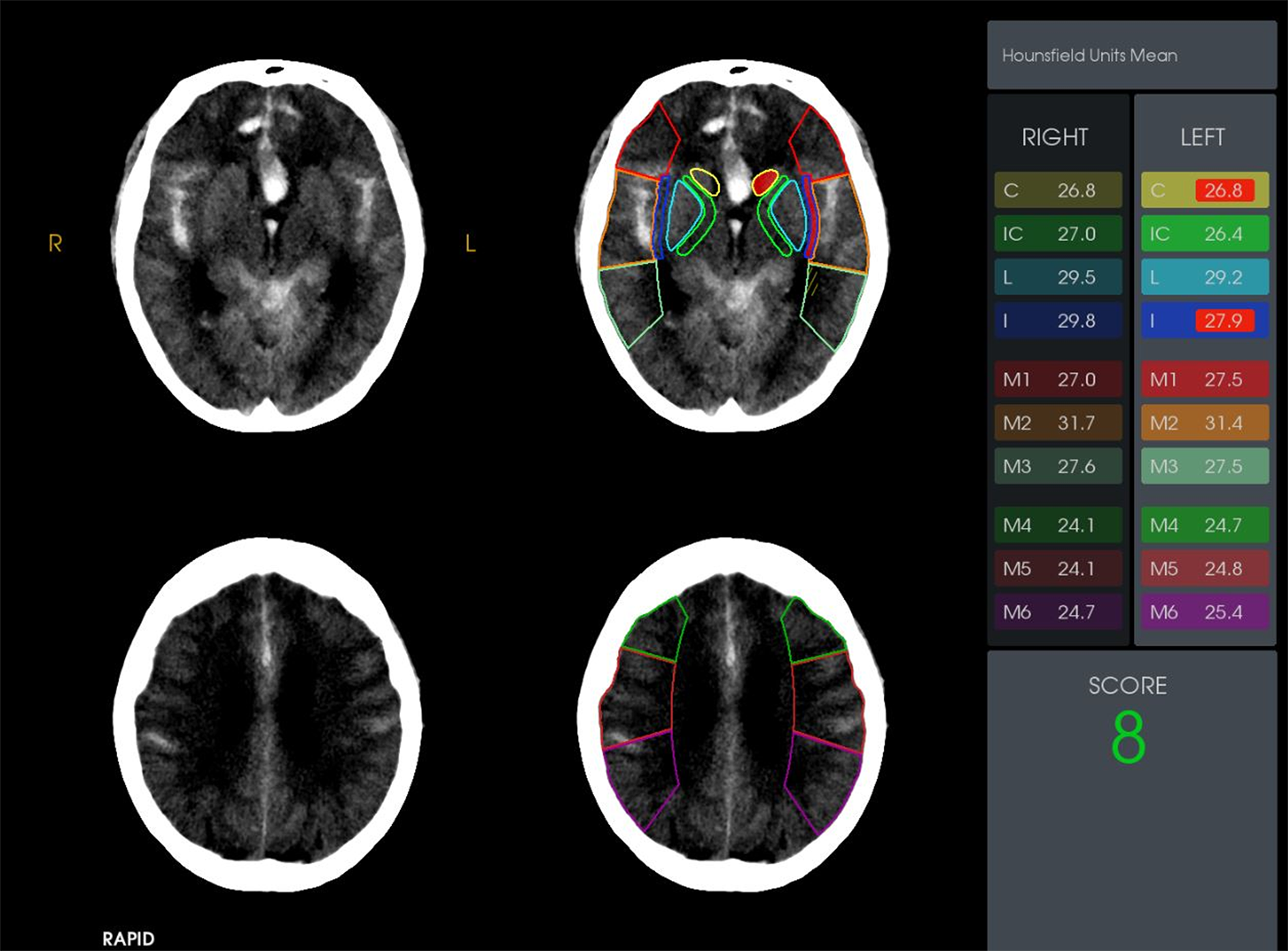

Supplement: Supplementary file 2 — Figure S2. [file ACN3-10-2373-s003.tif]
