# Supplementary material for: Insular cortex Hounsfield units predict postoperative neurocardiogenic injury in patients with aneurysmal subarachnoid hemorrhage
Source: Ann Clin Transl Neurol. 2023 Oct 18;10(12):2373–85. doi: 10.1002/acn3.51926 (PMC10723248; doi:10.1002/acn3.51926)
Supplement: Supplementary file 3 — Table S1. [file ACN3-10-2373-s005.docx]

**Table S1**. Patients’ characteristics and group comparisons before and after propensity score matching.

| Patient Characteristics | Before propensity score matching | | |
| --- | --- | --- | --- |
|  | right IC Hu<28.65 | right IC Hu>28.65 | *p*-value |
| No. of patients | 280 | 250 |  |
| **Demographics** |  |  |  |
| Age, years; mean±SD | 54.9±11.7 | 56.1±11.3 | 0.241 |
| Gender, female, n (%) | 95 (33.9) | 111 (44.4) | 0.014 |
| Current drinking, n (%) | 18 (6.4) | 21 (8.4) | 0.386 |
| Current smoking, n (%) | 31 (11.1) | 26 (10.4) | 0.803 |
| Hypertension, n (%) | 138 (49.3) | 132 (52.8) | 0.419 |
| Hyperlipidemia, n (%) | 8 (2.9) | 4 (1.6) | 0.331 |
| Diabetes mellitus, n (%) | 19 (6.8) | 11 (4.4) | 0.235 |
| History of heart disease, n (%) | 20 (7.1) | 17 (6.8) | 0.877 |
| **Preoperative condition** |  |  |  |
| WFNS grade 4-5, n (%) | 51 (18.2) | 61 (24.4) | 0.082 |
| mFS grade 3-4, n (%) | 141 (50.4) | 176 (70.4) | <0.001 |
| Graeb score 5-12, n (%) | 14 (5.0) | 20 (8.0) | 0.159 |
| SEBES score 3-4, n (%) | 109 (38.9) | 118 (47.2) | 0.055 |
| Acute hydrocephalus, n (%) | 102 (36.4) | 106 (42.4) | 0.16 |
| **Imaging index** |  |  |  |
| Left IC Hu; mean±SD | 27.5±2.4 | 29.9±2.8 | <0.001 |
| Posterior circulation aneurysm, n (%) | 34 (12.1) | 24 (9.6) | 0.349 |
| Aneurysm size ^a^, median (IQR) | 5 (4, 7) | 5 (4, 8) | 0.663 |
| **Treatment modality** |  |  | 0.275 |
| Endovascular treatment, n (%) | 124 (44.3) | 99 (39.6) |  |
| Surgical clipping, n (%) | 156 (55.7) | 151 (60.4) |  |
| **Postoperative complication** |  |  |  |
| NCI, n (%) | 27 (9.6) | 56 (22.4) | <0.001 |
| sME | N1 (22) | N2 (44) |  |
| aEP | N3 (11) | N4 (22) |  |

| Patient Characteristics | After propensity score matching | | | | | |  |
| --- | --- | --- | --- | --- | --- | --- | --- |
|  | right IC Hu<28.65 | | right IC Hu>28.65 | | *p*-value | |  |
| No. of patients | 129 | | 129 | |  | |  |
| **Demographics** |  |  |  |  | |  | |
| Age, years; mean±SD | 55.3±11.8 | | 55.6±12.1 | | 0.867 | |  |
| Gender, female, n (%) | 53 (41.1) | | 46 (35.7) | | 0.370 | |  |
| Current drinking, n (%) | 12 (9.3) | | 12 (9.3) | | ＞0.99 | |  |
| Current smoking, n (%) | 15 (11.6) | | 21 (16.3) | | 0.281 | |  |
| Hypertension, n (%) | 71 (55.0) | | 65 (50.4) | | 0.454 | |  |
| Hyperlipidemia, n (%) | 0 (0) | | 1 (0.8) | | 0.316 | |  |
| Diabetes mellitus, n (%) | 5 (3.9) | | 7 (5.4) | | 0.554 | |  |
| History of heart disease, n (%) | 7 (5.4) | | 9 (7.0) | | 0.606 | |  |
| **Preoperative condition** |  | |  | |  | |  |
| WFNS grade 4-5, n (%) | 34 (26.4) | | 28 (21.7) | | 0.382 | |  |
| mFS grade 3-4, n (%) | 98 (76.0) | | 71 (55.0) | | <0.001 | |  |
| Graeb score 5-12, n (%) | 12 (9.3) | | 7 (5.4) | | 0.233 | |  |
| SEBES score 3-4, n (%) | 57 (44.2) | | 53 (41.1) | | 0.615 | |  |
| Acute hydrocephalus, n (%) | 53 (41.1) | | 46 (35.7) | | 0.370 | |  |
| **Imaging index** |  | |  | |  | |  |
| Left IC Hu; mean±SD | 28.5±2.9 | | 28.9±2.1 | | 0.281 | |  |
| Posterior circulation aneurysm, n (%) | 15 (11.6) | | 12 (9.3) | | 0.542 | |  |
| Aneurysm size ^a^, median (IQR) | 5 (4, 7) | | 5 (4, 8) | | 0.753 | |  |
| **Treatment modality** |  | |  | |  | |  |
| Endovascular treatment, n (%) | 54 (41.9) | | 54 (41.9) | | ＞0.99 | |  |
| Surgical clipping, n (%) | 75 (58.1) | | 75 (58.1) | |  | |  |
| **Postoperative complication** |  |  |  |  | |  | |
| NCI, n (%) | 14 (10.9) | | 26 (20.2) | | 0.039 | |  |
| sME | N1 (12) | | N2 (19) | |  | |  |
| aEP | N3 (6) | | N4 (11) | |  | |  |

IC, insular cortex; Hu, hounsfield unit; SD, standard deviation; WFNS, World Federation of Neurosurgical Surgeons; mFS, modified Fisher; SEBES, subarachnoid hemorrhage early brain edema score; IQR, interquartile range; NCI, neurocardiogenic injury; sME, elevated serum myocardial enzyme levels; aEP, aberrant echocardiography presentation

^a^ unit of measurement: mm
