# Supplementary material for: Insular cortex Hounsfield units predict postoperative neurocardiogenic injury in patients with aneurysmal subarachnoid hemorrhage
Source: Ann Clin Transl Neurol. 2023 Oct 18;10(12):2373–85. doi: 10.1002/acn3.51926 (PMC10723248; doi:10.1002/acn3.51926)
Supplement: Supplementary file 4 — Table S2. [file ACN3-10-2373-s002.docx]

| Table S2. The special baseline characteristics of the patients with postoperative NCI, as well as the subcategories of each NCI. | | | | | | |
| --- | --- | --- | --- | --- | --- | --- |
| Patient ID | Right IC value | Left IC value | type of intervention | history of cardiac disease | NCI | NCI subtype |
| 1 | 29.4 | 25.4 | ET | Yes | Yes | sME |
| 2 | 27.0 | 25.7 | SC | No | Yes | BOTH |
| 3 | 25.6 | 25.8 | SC | Yes | Yes | sME |
| 4 | 26.6 | 26.0 | ET | No | Yes | aEP |
| 5 | 26.6 | 26.3 | ET | No | Yes | sME |
| 6 | 28.8 | 26.4 | ET | No | Yes | sME |
| 7 | 27.7 | 26.6 | ET | No | Yes | BOTH |
| 8 | 27.9 | 26.6 | ET | No | Yes | sME |
| 9 | 29.8 | 26.6 | SC | No | Yes | sME |
| 10 | 27.2 | 26.7 | SC | No | Yes | sME |
| 11 | 27.7 | 26.9 | ET | No | Yes | BOTH |
| 12 | 27.0 | 27.0 | ET | No | Yes | sME |
| 13 | 28.3 | 27.0 | ET | No | Yes | sME |
| 14 | 30.3 | 27.1 | ET | No | Yes | aEP |
| 15 | 27.8 | 27.4 | SC | No | Yes | aEP |
| 16 | 28.4 | 27.4 | ET | No | Yes | sME |
| 17 | 28.6 | 27.5 | ET | No | Yes | sME |
| 18 | 28.7 | 27.6 | ET | No | Yes | BOTH |
| 19 | 27.4 | 27.7 | ET | No | Yes | sME |
| 20 | 28.3 | 27.7 | SC | No | Yes | sME |
| 21 | 28.4 | 27.7 | ET | Yes | Yes | aEP |
| 22 | 27.5 | 27.8 | ET | No | Yes | sME |
| 23 | 27.8 | 27.8 | ET | Yes | Yes | sME |
| 24 | 29.5 | 27.8 | SC | No | Yes | sME |
| 25 | 28.7 | 27.9 | ET | No | Yes | aEP |
| 26 | 28.8 | 27.9 | ET | No | Yes | sME |
| 27 | 29.8 | 27.9 | ET | No | Yes | sME |
| 28 | 28.0 | 28.0 | ET | Yes | Yes | aEP |
| 29 | 28.1 | 28.0 | ET | No | Yes | sME |
| 30 | 28.7 | 28.1 | ET | No | Yes | BOTH |
| 31 | 28.9 | 28.1 | SC | Yes | Yes | aEP |
| 32 | 29.7 | 28.1 | SC | No | Yes | BOTH |
| 33 | 28.7 | 28.2 | ET | No | Yes | sME |
| 34 | 27.6 | 28.4 | SC | No | Yes | sME |
| 35 | 28.5 | 28.4 | ET | No | Yes | BOTH |
| 36 | 31.5 | 28.4 | SC | No | Yes | sME |
| 37 | 28.8 | 28.5 | SC | No | Yes | aEP |
| 38 | 30.0 | 28.5 | ET | No | Yes | sME |
| 39 | 31.2 | 28.5 | SC | No | Yes | BOTH |
| 40 | 30.3 | 28.7 | ET | No | Yes | sME |
| 41 | 28.4 | 28.8 | ET | No | Yes | BOTH |
| 42 | 29.6 | 29.0 | SC | No | Yes | sME |
| 43 | 30.4 | 29.0 | ET | No | Yes | sME |
| 44 | 38.2 | 29.0 | SC | No | Yes | sME |
| 45 | 31.2 | 29.1 | ET | No | Yes | sME |
| 46 | 29.4 | 29.2 | ET | No | Yes | sME |
| 47 | 29.9 | 29.2 | ET | No | Yes | sME |
| 48 | 30.2 | 29.2 | ET | No | Yes | sME |
| 49 | 29.1 | 29.3 | ET | Yes | Yes | BOTH |
| 50 | 29.2 | 29.4 | ET | No | Yes | aEP |
| 51 | 29.3 | 29.4 | ET | No | Yes | sME |
| 52 | 29.8 | 29.4 | SC | Yes | Yes | sME |
| 53 | 41.7 | 29.4 | SC | No | Yes | aEP |
| 54 | 28.8 | 29.6 | ET | No | Yes | sME |
| 55 | 29.2 | 29.6 | ET | No | Yes | sME |
| 56 | 30.6 | 29.7 | SC | No | Yes | sME |
| 57 | 28.0 | 29.8 | SC | No | Yes | BOTH |
| 58 | 28.8 | 29.8 | SC | No | Yes | sME |
| 59 | 29.3 | 29.8 | SC | No | Yes | sME |
| 60 | 28.6 | 29.9 | SC | No | Yes | aEP |
| 61 | 29.3 | 29.9 | ET | No | Yes | sME |
| 62 | 29.8 | 29.9 | ET | No | Yes | aEP |
| 63 | 30.0 | 29.9 | ET | Yes | Yes | BOTH |
| 64 | 30.8 | 29.9 | ET | No | Yes | BOTH |
| 65 | 29.8 | 30.0 | ET | No | Yes | sME |
| 66 | 31.5 | 30.1 | SC | No | Yes | sME |
| 67 | 26.1 | 30.2 | SC | No | Yes | sME |
| 68 | 30.2 | 30.3 | ET | No | Yes | sME |
| 69 | 30.3 | 30.3 | SC | No | Yes | sME |
| 70 | 35.7 | 30.4 | SC | Yes | Yes | BOTH |
| 71 | 28.2 | 30.8 | ET | No | Yes | sME |
| 72 | 29.5 | 31.1 | SC | No | Yes | aEP |
| 73 | 37.7 | 31.1 | SC | No | Yes | sME |
| 74 | 30.1 | 32.4 | SC | No | Yes | sME |
| 75 | 35.3 | 32.4 | ET | No | Yes | sME |
| 76 | 35.1 | 32.6 | ET | Yes | Yes | BOTH |
| 77 | 33.3 | 34.5 | SC | No | Yes | sME |
| 78 | 35.3 | 35.2 | SC | Yes | Yes | aEP |
| 79 | 35.0 | 35.3 | ET | Yes | Yes | BOTH |
| 80 | 35.3 | 35.6 | ET | No | Yes | sME |
| 81 | 35.4 | 35.9 | SC | No | Yes | aEP |
| 82 | 36.6 | 37.7 | ET | No | Yes | aEP |
| 83 | 38.2 | 38.2 | SC | No | Yes | aEP |
| SC, surgical clipping; ET, endovascular treatment; sME, elevated serum myocardial enzyme levels; aEP, aberrant echocardiography presentation | | | | | | |
| "BOTH" represents both sME and aEP. | | | | | | |
