# Supplementary material for: Insular cortex Hounsfield units predict postoperative neurocardiogenic injury in patients with aneurysmal subarachnoid hemorrhage
Source: Ann Clin Transl Neurol. 2023 Oct 18;10(12):2373–85. doi: 10.1002/acn3.51926 (PMC10723248; doi:10.1002/acn3.51926)
Supplement: Supplementary file 5 — Table S3. [file ACN3-10-2373-s004.docx]

**Table S3.** The comparisons of patients’ special characteristics between Right IC Hu groups before and after propensity score matching.

| Patient Characteristics | Before propensity score matching | | |
| --- | --- | --- | --- |
|  | right IC Hu<28.65 | right IC Hu>28.65 | *p*-value |
| No. of patients | 280 | 250 |  |
| **Demographics**  History of heart disease, n (%) | 20 (7.1) | 17 (6.8) | 0.877 |
| **Treatment modality** |  |  | 0.275 |
| Endovascular treatment, n (%) | 124 (44.3) | 99 (39.6) |  |
| Surgical clipping, n (%) | 156 (55.7) | 151 (60.4) |  |
| **Postoperative complication** |  |  |  |
| NCI, n (%) | 27 (9.6) | 56 (22.4) | <0.001 |
| sME | N1 (22) | N2 (44) |  |
| aEP | N3 (11) | N4 (22) |  |

| Patient Characteristics | After propensity score matching | | | | | |  |
| --- | --- | --- | --- | --- | --- | --- | --- |
|  | right IC Hu<28.65 | | right IC Hu>28.65 | | *p*-value | |  |
| No. of patients | 129 | | 129 | |  | |  |
| **Demographics** |  |  |  |  | |  | |
| History of heart disease, n (%) | 7 (5.4) | | 9 (7.0) | | 0.606 | |  |
| **Treatment modality** |  | |  | |  | |  |
| Endovascular treatment, n (%) | 54 (41.9) | | 54 (41.9) | | >0.99 | |  |
| Surgical clipping, n (%) | 75 (58.1) | | 75 (58.1) | |  | |  |
| **Postoperative complication** |  |  |  |  | |  | |
| NCI, n (%) | 14 (10.9) | | 26 (20.2) | | 0.039 | |  |
| sME | N1 (12) | | N2 (19) | |  | |  |
| aEP | N3 (6) | | N4 (11) | |  | |  |

IC, insular cortex; Hu, hounsfield unit; NCI, neurocardiogenic injury; sME, elevated serum myocardial enzyme levels; aEP, aberrant echocardiography presentation
